# Hypertrophy of unaffected cardiomyocytes correlates with severity of cardiomyopathy in female patients with Fabry disease

**DOI:** 10.1186/s13023-021-01803-7

**Published:** 2021-04-10

**Authors:** Cristina Chimenti, Romina Verardo, Andrea Frustaci

**Affiliations:** 1 Department of Clinical, Internal, Anesthesiologist and Cardiovascular Sciences, Sapienza University, Viale del Policlinico 155, 00161 Rome, Italy; 2 Cellular and Molecular Cardiology Lab, IRCCS L. Spallanzani, Rome, Italy

**Keywords:** Fabry Disease, Gene mutation, Endomyocardial biopsies

## Abstract

**Aim:**

To investigate the contribution of unaffected cardiomyocytes in Fabry disease cardiomyopathy.

**Findings:**

Left ventricular (LV) endomyocardial biopsies from twenty-four females (mean age 53 ± 11 ys) with Fabry disease cardiomyopathy were studied. Diagnosis of FD was based on the presence of pathogenic GLA mutation, Patients were divided in four groups according with LV maximal wall thickness (MWT): group 1 MWT ≤ 10.5 mm, group 2 MWT 10.5–15 mm, group 3 MWT 16–20 mm, group 4 MWT > 20 mm. At histology mosaic of affected and unaffected cardiomyocytes was documented. Unaffected myocytes’ size ranged from normal to severe hypertrophy. Hypertrophy of unaffected cardiomyocytes correlated with severity of MWT (p < 0.0001, Sperman r 0,95). Hypertrophy of unaffected myocytes appear to concur to progression and severity of FDCM. It is likely a paracrine role from neighboring affected myocytes.

Fabry disease (FD) is an X‐linked inborn error of glycosphingolipid catabolism caused by deleterious mutations in the α‐galactosidase A (GLA) gene encoding for a lysosomal hydrolase deputed to the catabolism of neutral glycosphingolipids [1, 2]. The marked deficiency or absence of GAL activity results in the systemic accumulation of globotriaosylceramide and related glycosphingolipids within the lysosomes, particularly in microvascular endothelial cells, vascular smooth muscle cells, renal tubular cells, podocytes, and cardiomyocytes [3–5]. The disease can manifest in a classic form or in late onset form, involving only kidney (renal variant) or heart (cardiac variant). Cardiac variant of FD has been described in up to 6% of men and 12% of women with late-onset hypertrophic cardiomyopathy (HCM) [6, 7]. Cardiac involvement, consisting of progressive left ventricular hypertrophy (LVH), is very common and is the most frequent cause of death.

FD cardiomyopathy (CM) is expected to be less severe in female than in male patients because of the X-linkage and skewed X chromosome inactivation. Nevertheless this is often not the case and the reason is still unclear.

We investigated the possible contribute of unaffected cardiomyocytes to severity of FDCM in female patients.

Left ventricular (LV) endomyocardial biopsies from twenty-four females (mean age 53 ± 11 ys) with FDCM were studied. Diagnosis of FDCM was based on the presence of pathogenic GLA mutation in female members of **11** families with different age and severity of cardiac involvement, and accumulation of glycosphyngolipids in the cardiac cells manifesting as myelin bodies at ultrastructural examination of glutaraldehyde-fixed endomyocardial samples. Patients were divided in four groups according with LV maximal wall thickness (MWT): group 1 MWT ≤ 10.5 mm, group 2 MWT 10.5–15 mm, group 3 MWT 16–20 mm, group 4 MWT > 20 mm. Clinical, ecocardiographic and pathologic characteristics of patients are reported in Table 1. No systemic artery hypertension nor cardiac valve disease causing volume/pressure overload of LV chamber were registered in our cohort. In each patient percent of non-affected cells, diameter at nuclear level of affected and non-affected cardiomyocytes and percent area of vacuoles in affected cells were morphometrically evaluated at histology. Cardiomyocyte diameter ≤ 15 micron was considered normal. Data were correlated with MWT at echocardiography.

**Table 1:** Clinical and echocardiographic characteristics of female patients with FD cardiomyopathy

	Group 1 n = 4	Group 2 n = 9	Group 3 n = 8	Group 4 n = 3
Age	38 ± 3	51.4 ± 6.7	60.3 ± 9.6	63.0 ± 5.6
**Cardiac symptoms**				
Angina,%	0	33	63	33
Dyspnea,%	0	67	87	100
Palpitation, %	100	100	100	100
**Ecocardiography**				
Maximal wall thickness, mm	9.03 ± 0.5	13.3 ± 1.5	17.1 ± 1.2	21.0 ± 1.7
Left ventricular ejection fraction,%	65 ± 7	66 ± 3	64 ± 3	62 ± 5
E/A ratio	1.2. ± 0.4	1. ± 0.4	0.9 ± 0.7	0.9 ± 0.3
**Morphometry**				
Percent of non affected cardiomyocytes	30.5 ± 4.2	32.4 ± 12.5	32.8 ± 9.6	32.7 ± 3.3
Diameter of non affected cardiomyocytes, micron	13.3 ± 3.3	21.0 ± 2.5	26.5 ± 2.5	33.7 ± 3.4
Diameter of affected cells, micron	26.2 ± 9.1	37.8 ± 18.6	43.3 ± 20.1	51.7 ± 14.9
Percent area occupied by vacuoles	56.6 ± 65.7	62.2 ± 69.5	65.2 ± 72.8	67.1 ± 58.9
Fibrosis, %	2.4 ± 5.7	8.3 ± 12.7	14.4 ± 19.0	17.0 ± 5.3
Genetic	c.680G > A, c.335G > A, c.668G < A	c.680G > A, cN46delG, c.548G > G, c.666delC, c.901C > G, c.644A > G	c.C835C > A, c668G < A, c547 + 1G > A, c.644A > G	c.679C > T, c.335G > A, c.644A > G

Overall percent of non-affected cardiomyocytes was 32 ± 41% and was similar among the 4 groups (Table 1). Each specimen showed the presence of mosaic of affected and unaffected cells. Differentiation between affected and unaffected cells has been supported by histology, including Sudan Black staining, immunofluorescence for antiGB3 antibodies on frozen sections, and electronmicroscopy. Mean value of cardiomyocyte diameter of unaffected cells increased with the severity of left ventricular hypertrophy (*p* < 0.0001, Sperman r 0.95 Fig. 1). In particular, in Group 1 unaffected cardiomyocytes were within normal limits (Fig. 1a). In Group 2 and in Group 3 the diameter of unaffected cells increased progressively (Fig. 1b, c). In patients with remarkable increase in MWT non-affected cells were severely hypertrophied and focally disarrayed (Fig. 1d). Affected cells were enlarged in all patients (mean 40.1 ± 48 micron) with percent vacuoles ranging from 40 to 75% of the cell area. Affected cardiomyocytes area statistically significant correlates with MWT (*p* = 0.02, Sperman r = 0.5).

**Fig. 1:**
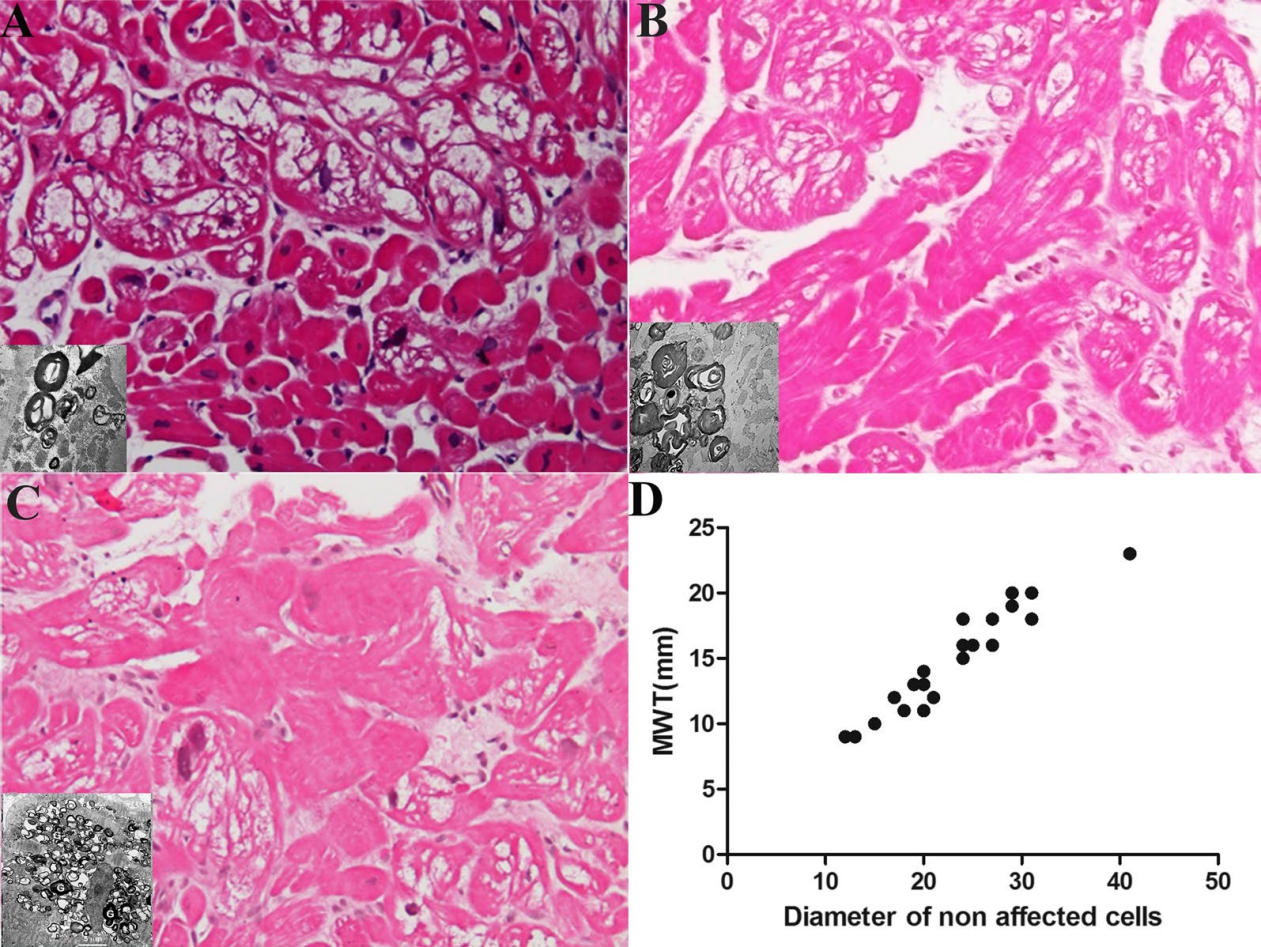
**a** Mosaic with normal and affected myocytes. (H&E 200×) in a female with pre-hypertrophic Fabry Cardiomyopathy (10.3 mm LVMWT and preserved EF (58%). Insert shows accumulation of glycolipids in the form of myelin bodies. **b** Moderate hypertrophy of unaffected myocytes in Fabry female with moderate LV hypertrophy (17 mm LV MWT and EF 55%). **c** Severe hypertrophy with disarray of unaffected myocytes interspersed with enlarged vacuolated cells. (H&E ×200) in a Fabry female with severe LV hypertrophy (LV MWT 22 mm, Left Bundle Branch Block, AV block, implanted Pace-Maker, EF 30%, died because of heart failure). Insert shows accumulation of glycolipids in the form of myelin bodies. **d** correlation between MWT and diameter of non affected cells showing a linear correlation

Major sarcomeric genes involved in hypertrophic cardiomyopathy including β-myosin heavy chain, cardiac myosin binding protein C, regulatory myosin light chain, cardiac troponin T, cardiac troponin I and actin were tested and showed no mutations.

FD cardiomyopathy although characterized by cell mosaic including affected and unaffected myocytes may have the same degree of LV hypertrophy and manifest unfavorable evolution as in male where all cardiomyocytes are equally ill [8]. The first explanation to this occurrence is an unbalanced cell ratio, because of skewed inactivation of X chromosome, favoring the development of affected cardiomyocytes. Indeed, in our study unaffected cells represented overall 32% of all myocytes and was similar among groups, suggesting the number of affected cells could not explain the different degree of LV hypertrophy. Furthermore, in our cohort no sarcomere gene anomalies have been documented in association to GLA mutations to justify the different degree of LV thickening.

Conversely, the present study suggests hypertrophy of unaffected cardiomyocytes to be a major determinant of LV hypertrophy in female with FDCM. It increases with progression of the disease and correlates with severity of cardiomyopathy. To this regard there is general agreement on the glycolipid secretion, in particular lysoGB3, in the extracellular space from Fabry cells, determining a hypertrophic stimulus to the neighboring unaffected cardiomyocytes [9, 10]. Indeed, increased circulating levels of lyso-Gb3 have been demonstrated also in females with normal enzymatic activity [11]. Progressive accumulation of Gb3 into affected cardiomyocytes would be followed by increasing interstitial amount of Gb3 (partly implemented by cell necrosis with release of glicolipids) with enhanced hypertrophic activation of unaffected cells.

However several alternative hypotheses can be considered in the mechanism of hypertrophy of unaffected cells, including increased left ventricular end-diastolic pressure, activation of renin-angiotensin system, epigenetic/environmental factors and additional mutations not known / found in the genetic study. In particular in unaffected cells a permissive expression from both alleles, with enough enzyme from the normal allele to prevent formation of myelin bodies, but not enough to stop hypertrophy [11] cannot be excluded.

In conclusion, hypertrophy of unaffected cardiomyocytes appears to concur to progression and severity of FDCM in females. It is likely a paracrine role from neighboring affected myocytes.

However, give the cross-sectional observational characteristic of this study obtained on very few patients, we cannot draw conclusions from it, and further studies are needed to confirm our hypothesis.

## Data Availability

The datasets used and analyzed during the current study are available from the corresponding author on reasonable request.

## Abbreviations

FD: Fabry disease
CM: Cardiomyopathy
LV: Left ventricular
MWT: Maximal wall thickness
